# Try or Die: Dynamics of Plant Respiration and How to Survive Low Oxygen Conditions

**DOI:** 10.3390/plants11020205

**Published:** 2022-01-13

**Authors:** Jay Jethva, Romy R. Schmidt, Margret Sauter, Jennifer Selinski

**Affiliations:** 1 Department of Plant Developmental Biology and Plant Physiology, Faculty of Mathematics and Natural Sciences, Botanical Institute, Christian-Albrechts University, D-24118 Kiel, Germany; jjethva@bot.uni-kiel.de (J.J.); msauter@bot.uni-kiel.de (M.S.); 2 Department of Plant Biotechnology, Faculty of Biology, University of Bielefeld, D-33615 Bielefeld, Germany; romy.schmidt@uni-bielefeld.de; 3 Department of Plant Cell Biology, Botanical Institute, Faculty of Mathematics and Natural Sciences, Christian-Albrechts University, D-24118 Kiel, Germany

**Keywords:** chloroplasts, energy metabolism, hypoxia, mitochondria, redox, re-oxygenation

## Abstract

Fluctuations in oxygen (O_2_) availability occur as a result of flooding, which is periodically encountered by terrestrial plants. Plant respiration and mitochondrial energy generation rely on O_2_ availability. Therefore, decreased O_2_ concentrations severely affect mitochondrial function. Low O_2_ concentrations (hypoxia) induce cellular stress due to decreased ATP production, depletion of energy reserves and accumulation of metabolic intermediates. In addition, the transition from low to high O_2_ in combination with light changes—as experienced during re-oxygenation—leads to the excess formation of reactive oxygen species (ROS). In this review, we will update our current knowledge about the mechanisms enabling plants to adapt to low-O_2_ environments, and how to survive re-oxygenation. New insights into the role of mitochondrial retrograde signaling, chromatin modification, as well as moonlighting proteins and mitochondrial alternative electron transport pathways (and their contribution to low O_2_ tolerance and survival of re-oxygenation), are presented.

## 1. Introduction

The increasing frequency of floods resulting from severe rainfall, a rise in the seawater level, and the glacial melt caused by climate change, has devastating effects on plant growth and productivity [1,2,3,4]. As a result of restricted gas diffusion underwater, flooded plants experience dramatic variations in molecular oxygen (O_2_) availability, ranging from partial O_2_ deficiency (hypoxia; usually between 1% and 5% O_2_) to total O_2_ depletion (anoxia, 0% O_2_). Two types of hypoxia can be distinguished: environmental hypoxia can be caused by specific environmental cues, while developmental hypoxia can also occur in tissues and organs under normal O_2_ availability. On the one hand, environmental hypoxia results from soil waterlogging, which occurs when only the root zone is flooded and cannot respire due to excessive water in the soil [5]. Under these conditions, nutrient uptake via the root zone and stomata conductance, as well as CO_2_ assimilation in aerial parts of the plant, are reduced. In contrast to terrestrial plants, wetland plants tolerate longer and stronger hypoxic conditions through a combination of escape (increased metabolism and growth) and quiescence (slowing growth and saving metabolic resources) strategies [6]. On the other hand, environmental hypoxia is generated by submergence. In this case, the entire plant (roots and aerial parts of the plant) is under water [5], which may, similarly to wetland plants, trigger quiescence or escape strategies as well as ethylene entrapment [7,8]. However, hypoxic and anoxic conditions even occur during normal plant growth and development. Hence, O_2_ concentrations differ in plant tissues and plant species. Since the anatomy of specific tissues or organs, such as seeds, fruits and roots, may limit O_2_ diffusion due to dense tissue, hypoxic conditions can occur [9,10,11]. For instance, the O_2_ concentrations in the centers of growing potato tubers can decrease to around 5% [12], while O_2_ is undetected in seeds of barley (*Hordeum vulgare* L.) and pea (*Pisum sativum* L.) [9,10,13,14]. Most plant organs possess a relatively high surface-to-volume ratio, allowing for efficient O_2_ diffusion, thereby preventing the occurrence of hypoxic conditions [9]. However, due to high metabolic activity, even tissues in which O_2_ diffusion is unlimited may become hypoxic. For example, high local rates of O_2_ consumption have been shown to occur in the phloem of castor bean (*Ricinus communis* L.), which reaches O_2_ levels of about 7% [9], and in the shoot apical meristem of Arabidopsis and *Solanum lycopersicum,* which shows O_2_ levels below 5% [15]. Furthermore, an O_2_ gradient is formed within developing monocot anthers as a result of rapid cell proliferation, which has been shown using *Zea mays* mutants (*Zmmsca1, male sterile converted anther1*), characterized by an early developmental defect after anther primordial formation [7,16].

During evolution, plants have developed several morphological and physiological adaptations that allow for plant survival during environmentally low O_2_ conditions [17,18,19]. The anatomical alterations of plants grown under these conditions include the formation of lateral roots (e.g., *Arabidopsis thaliana*), root aerenchyma or adventitious roots (e.g., *Zea mays*, *Oryza sativa*), and the elongation of the coleoptile (e.g., *Oryza sativa*, *Echinochloa* spp.), internode (e.g., deepwater rice) or petiole (e.g., *Rumex palustris*) to re-establish gas exchange [20]. Furthermore, plants can limit their growth to save carbohydrate and energy reserves until O_2_ becomes available again in post-hypoxic or anoxic conditions [21]. On the physiological level, limitations in O_2_ availability lead to restrictions of aerobic metabolism. Therefore, plants have to adapt their metabolism in order to guarantee sufficient energy supply for survival, but at the expense of growth. Under low O_2_ conditions, ATP generation via oxidative phosphorylation (OXPHOS) in plant mitochondria, as well as the regeneration of NAD^+^ via the mitochondrial electron transport chain (mtETC), are reduced. Under these conditions, the anaerobic metabolism is upregulated instead, in which ATP and NAD^+^ supplies solely depend on glycolytic fermentation. While aerobic metabolism generates 36–38 molecules ATP per molecule glucose, anaerobic metabolism generates 2 to 3 molecules ATP only. Hence, the cellular ATP content is extremely low under O_2_ deficiency [19]. The re-routing of plant energy metabolism under limited O_2_ conditions ultimately leads to cell acidification and the accumulation of reducing equivalents, causing excess generation of reactive oxygen species (ROS) and reactive nitrogen species (RNS) that potentially lead to cell damage [22,23].

To prevent cell damage under low-O_2_ conditions, plant cells execute various stress responses, including major metabolic re-routing, hormonal regulation, changes in mitochondrial biogenesis, as well as the reprograming of gene expression [24,25,26,27,28,29,30,31,32,33,34,35]. The latter includes the upregulation of transcripts that are required to meet the energy demands of plant cells via fermentative glycolysis, such as alcohol dehydrogenase (ADH), pyruvate decarboxylase (PDC) and alanine aminotransferase (ALAT), while downregulation of gene transcription associated with ATP-consuming pathways, such as lipid metabolism, secondary metabolism, transport, signaling, and redox regulation, is initiated [15,30,31,36,37]. During fermentative glycolysis, ethanol and lactate accumulation (catalyzed by ADH, PDC and lactate dehydrogenase (LDH)) can reach toxic levels in plant cells. However, comparative analyses of flood-tolerant and flood-sensitive plant species have demonstrated that tolerance to flooding is related to ethanol tolerance in plants [38].

While a lot of information has been published concerning physiological and molecular events occurring during hypoxia, there is still a big gap in our knowledge about re-oxygenation in post-hypoxic conditions. Post-hypoxic and -anoxic stresses as experienced during re-oxygenation and simultaneous re-illumination cause photoinhibition that is accompanied by an increased generation of excess ROS and RNS, which leads to cell damage. To counteract this, the aerobic metabolism is suppressed during recovery from O_2_ deficiency, leading to an inhibition of metabolic functions [39,40,41,42].

The present review aims to update current knowledge about mitochondrial mechanisms enabling plants to adapt to low-O_2_ stress and re-oxygenation post hypoxia. We will summarize timely relevant research that has been carried out to reveal plant mitochondrial strategies to survive low O_2_ concentrations as well as re-oxygenation. In this context, we will focus on the metabolic, transcriptional as well as post-translational levels.

## 2. Re-Routing of Plant Energy Metabolism as a Strategy to Survive O_2_ Deprivation

**Figure 1:**
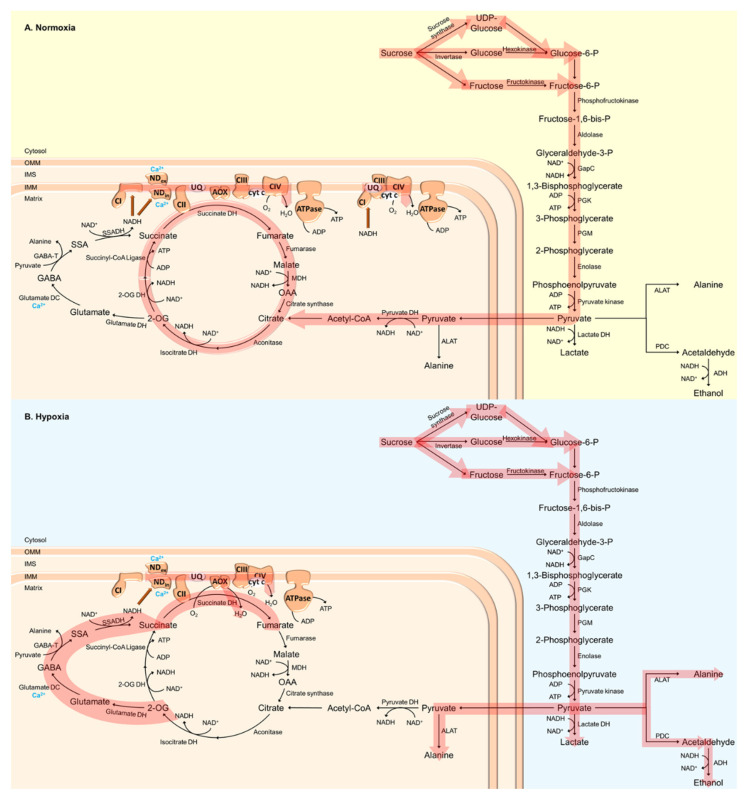
Schematic visualization of plant respiratory metabolism under (**A**) normoxic and (**B**) hypoxic conditions. Under low O_2_ conditions, ATP generation via oxidative phosphorylation (OXPHOS) in plant mitochondria, as well as regeneration of NAD^+^ via the mitochondrial electron transport chain (mtETC), are reduced. Under these conditions, the anaerobic metabolism is upregulated instead, in which ATP and NAD^+^ supply solely depend on glycolytic fermentation. 2-OG, 2-oxoglutarate; 2-OG DH, 2-oxoglutarate dehydrogenase; ADH, alcohol dehydrogenase; ALAT, alanine aminotransferase; AOX, alternative oxidase; CI, complex I (NADH dehydrogenase); CII, complex II (succinate dehydrogenase); CIII, complex III (cytochrome bc_1_ complex); CIV, complex IV (cytochrome c oxidase, COX); cyt c, cytochrome c; GABA, γ-aminobutyric acid; GABA-T, GABA-transaminase; GapC, cytosolic glyceraldehyde-3-phosphate dehydrogenase; glutamate DC, glutamate decarboxylase; glutamate DH, glutamate dehydrogenase; IMM, inner mitochondrial membrane; IMS, intermembrane space; isocitrate DH, isocitrate dehydrogenase; lactate DH, lactate dehydrogenase; MDH, malate dehydrogenase; NDin/ex, internal/external alternative NAD(P)H dehydrogenase; OAA, oxaloacetate; OMM, outer mitochondrial membrane; PDC, pyruvate decarboxylase; PGK, phosphoglycerate kinase; PGM, phosphoglycerate mutase; pyruvate DH, pyruvate dehydrogenase; SSA, succinic semialdehyde; SSADH, succinic semialdehyde dehydrogenase; succinate DH, succinate dehydrogenase; UQ, ubiquinone.

The energetic requirements of plants are fulfilled by photosynthesis and respiration, the latter supplying 100% of the ATP required in darkness, non-green or heterotrophic tissues. Respiration comprises glycolysis, the tricarboxylic acid (TCA) cycle, and the mtETC (Figure 1). It provides metabolites and plays a role in the maintenance of photosynthesis. Even in green tissues, respiration is essential. Respiration is responsive to abiotic and biotic stresses, demonstrating its crucial role in plant stress acclimation to adverse growth conditions [43]. It can vary dramatically depending on plant species, tissue and growth conditions [44,45]. Therefore, plant respiration represents an important hub, linking energy generation for growth and stress tolerance. Plant respiration is highly flexible, to meet the varying demands of plant growth and defense, especially under low O_2_ conditions.

A major factor affecting plant respiration is O_2_ depletion, since O_2_ is the final electron acceptor in the mtETC [46]. Low O_2_ conditions have been shown to inactivate OXPHOS and inhibit mtETC terminal oxidase (cytochrome c oxidase, COX) (Figure 1) [47,48,49,50,51]. To circumvent an energy crisis and the excess generation of ROS at complexes I and III, plants switch from aerobic to anaerobic metabolism (fermentation) to generate ATP through glycolysis and to regenerate NAD^+^ through ethanolic fermentation (Figure 1) [19]. In the cytosol, pyruvate is reduced to lactate by LDH, thus maintaining the redox balance without the loss of carbon that is associated with alcoholic fermentation. However, the accumulation of lactate in turn leads to a decrease in pH in the cytosol that deactivates LDH and activates PDC [52]. PDC catalyzes the decarboxylation of pyruvate, thereby generating acetaldehyde that is finally reduced to ethanol by ADH (Figure 1) [53]. Under low O_2_ conditions, the increased expression and activity of ADH as well as ethanol accumulation could be observed (Figure 1) [28,54,55]. The overexpression of *PDC* and *ADH* in Arabidopsis has been shown to promote tolerance against O_2_ deficiency [56]. In contrast, *adh* and *pdc* loss-of-function mutants of maize, rice and Arabidopsis are more sensitive towards O_2_ deprivation, indicating the importance of ethanolic fermentation for low-O_2_ stress tolerance [37,57,58,59]. In addition to fermentation, the Pasteur effect contributes to plant survival under low O_2_ conditions by inhibiting OXPHOS. Thereby, carbohydrate metabolism is accelerated and energy can be maintained [60,61,62].

An even higher flexibility of plant metabolism can be reached by the action of the TCA cycle. For instance, ALAT directly links glycolysis and the TCA cycle. The interconversion of 2-oxoglutarate (2-OG) to succinate catalyzed by ALAT generates NADH, which is in turn used by malate dehydrogenase (MDH) to reduce oxaloacetate (OAA) to malate (Figure 1). Together with succinate-coenzyme A (CoA) ligase, these enzymes contribute to the generation of ATP under hypoxic conditions [19,63]. As mentioned above, various enzymes involved in the TCA cycle increase plant survival under low O_2_ conditions, leading to an increased concentration of fumarate, aconitate, citrate and succinate in wheat and rice [55,64,65]. Furthermore, hypoxia has been demonstrated to promote malate, γ-aminobutyric acid (GABA) and alanine biosynthesis (Figure 1B) [66]. O_2_ deprivation leads to a rapid accumulation of GABA, as has been shown in various plant species [67,68,69,70,71,72]. GABA synthesis is stimulated by low O_2_ conditions due to increased cytosolic calcium (Ca^2+^) concentrations and the activation of glutamate decarboxylase by the calmodulin (CaM)-dependent pathway (Figure 1B) [73]. However, GABA can also be generated via polyamine degradation or the decarboxylation of proline [74,75]. In the mitochondrial matrix, GABA can be catabolized by GABA transaminase, leading to the generation of succinic semi-aldehyde and alanine (Figure 1) [74,76]. To date, the physiological role of hypoxia-induced GABA production is unknown. However, the GABA shunt has been suggested as an important adaptive mechanism to store carbon and nitrogen that would otherwise be lost under O_2_ deprivation [35,77,78]. Furthermore, the GABA shunt could be responsible for the maintenance of osmotic potential, the prevention of ROS accumulation and the inhibition of cell death [79,80]. Interestingly, GABA accumulation has also been hypothesized to be involved in the regulation of cytosolic pH and increased energy generation via the activation of the GABA shunt and the TCA cycle [73,81]. Recently, Wu et al. [82] demonstrated that a hypoxia-induced increase in GABA levels is essential for the restoration of the membrane potential, and to prevent ROS-induced disturbances to ion homeostasis.

About 50 years ago, the first reports were published describing mitochondrial degeneration and developmental failure during O_2_ deprivation that could be reversed upon re-oxygenation [83,84,85,86,87]. Interestingly, the dimerization and subsequent oligomerization of the ATP synthase has been demonstrated to contribute to cristae formation, thereby directly linking mitochondrial morphology and supercomplex formation in the mtETC in plants [88]. The function of supercomplexes for respiratory electron transport is still puzzling, but they have been hypothesized to (i) increase the stability of individual complexes, (ii) affect membrane structure and increase the dense packing of proteins within the membranes, (iii) channel electrons between the reactive sites of the mtETC complexes within supercomplexes, and (iv) affect the direction of electron flow from and towards the various components of alternative mtETC (alternative NAD(P)H dehydrogenases (NDs) and alternative oxidase (AOX)), which would enable the fine-tuning of plant energy metabolism and ATP yield under adverse growth conditions such as hypoxia [89,90,91,92,93,94]. However, Ramírez-Aguilar et al. [95] demonstrated that, under low O_2_ conditions, complex I dissociates from the respirasomes to support electron transfer from cytosolic NAD(P)H to the mtETC via NDs (Figure 1). Cell acidification and the accumulation of organic acids, as found during hypoxia, inhibit complex I activity and activate NDs. Thereby, the oxidation of cytosolic NADH shifts from complex I to the NDs (Figure 1) to keep glycolysis running when OXPHOS is reduced during O_2_ deprivation [96,97,98].

While extensive research has been carried out using roots or whole seedlings kept in darkness under hypoxic or anoxic conditions, knowledge about the effect of O_2_ deprivation on photosynthetic tissues or organs such as leaves and shoots is scarce. However, distinct responses of roots and shoots to limited O_2_ availability have been identified [35,99,100,101,102,103,104,105]. While roots exhibit a typical hypoxic response, such as the accumulation of GABA and alanine as well as a strong decrease in raffinose, sucrose, hexoses, and pentoses, leaves exhibit a strong increase in starch, sugars, sugar derivatives, and phenolics [105]. In addition, decreased expressions of nitrite reductase and declines in several amino acids could be observed in the leaves, but not the roots, of waterlogged plants [106,107,108]. Together, these data indicate that limited O_2_ availability leads to the inhibition of sugar export and N assimilation. Furthermore, low O_2_ conditions induce a decrease in stomatal conductance, a reduced leaf area, leaf senescence and lower chlorophyll content, which altogether lead to a rapid decrease in the photosynthetic rate [109,110,111,112,113]. However, shoots with access to partial photosynthesis or dissolved O_2_ are less perturbed, but still express hypoxia-responsive genes and perform anaerobic metabolism [35].

## 3. Signaling Events Initiating Plant Hypoxic Responses

Second messengers, such as ROS and RNS, as well as Ca^2+^, have been widely discussed to play an important role under adverse growth conditions [114,115]. Since most cellular functions are compromised under hypoxic conditions that lead to cytosolic acidification and the elevation of cytosolic Ca^2+^ concentrations, as well as the accumulation of ROS and RNS [22,23], these factors may be considered as signal transducers when O_2_ availability is limited.

### 3.1. The Cytosolic pH Decreases during Hypoxia and Promotes Anaerobic Metabolism

Low O_2_ conditions lead to a decrease in cytosolic pH, which drops from a pH value of 7.5 under normoxia to pH 6.0 during hypoxia [116]. While cytosolic acidification provides optimal conditions for anaerobic metabolism, aerobic metabolism is suppressed [117,118]. The origin of cytosolic acidification remains controversial. However, different mechanisms have been suggested to contribute to cytosolic acidification: (i) the switch to ethanol production and (ii) accumulation of lactate generated via anaerobic carbohydrate catabolism, (iii) ATP depletion, which reduces the activity of the plasma membrane and tonoplast proton pumps, (iv) induced membrane depolarization leading to potassium (K^+^) loss via K_out_^+^ channels, and (v) the inhibition of K^+^ uptake into root tip cells [119,120,121,122]. 

### 3.2. The Rapid Increase in Cytosolic Ca^2+^ Concentrations Enables Adaptation to Low O_2_ Conditions

Ca^2+^ is one of the most important ubiquitous intracellular second messengers that are involved in several signal transduction pathways in plants. Free cytosolic Ca^2+^ concentrations increase in response to various physiological stimuli of an abiotic and biotic nature [123,124,125]. A rapid increase in cytosolic Ca^2+^ concentrations in response to low O_2_ conditions has been observed in several plant species [116,126,127,128,129]. Ca^2+^ waves that are generated and spread during hypoxia result in the activation of several enzymes and genes, enabling adaptive responses at the cellular, tissue and plant levels [130,131,132]. For instance, Ca^2+^ is involved in the activation of glutamate decarboxylase (initiation of the GABA shunt), as well as internal and external NDs in plant mitochondria (Figure 1). Interestingly, cytosolic acidification has been demonstrated to be an important activator of external NADPH oxidation, by decreasing the Ca^2+^ requirements for external NDs [133]. Glutamate decarboxylase catalyzes the interconversion of glutamate to GABA (Figure 1). Its Ca^2+^-dependent activation therefore leads to GABA accumulation, causing a shift from lactate to alanine formation, which in turn leads to the stabilization of the cytosolic pH under hypoxic conditions [80,134]. On the transcript level, Ca^2+^ initiates transcription of *ADH* and *PDC,* which are both involved in hypoxic fermentation [126].

### 3.3. Reactive Oxygen and Nitrogen Species Accumulate during Hypoxia

Low O_2_ stress affects respiration, and has a severe impact on mitochondrial metabolism, the ATP/ADP ratio, the NAD(P)H redox state, the proton motive force, the antioxidant pool, as well as ROS/RNS homeostasis, which initiates mitochondrial retrograde responses [135,136,137,138]. Under hypoxic conditions, ROS accumulation occurs in mitochondria due to the lack of electron acceptors in the mtETC and the apoplast via respiratory burst oxidase homologs (RBOHs) that belong to the group of NADPH oxidases [139]. In mitochondria, limited O_2_ conditions activate the so-called ROS burst, as well as mitogen-activated protein kinases 3 and 6 (MPK3 and MPK6) [104]. Furthermore, mitochondria-generated hydrogen peroxide (H_2_O_2_) can cross the mitochondrial membrane and can be released into the cytosol [140]. In the apoplast, NADPH oxidases catalyze the electron flow from NADPH to O_2_. Thereby, superoxide anions are generated that are rapidly converted to H_2_O_2_ [141]. Torres et al. [142] showed that superoxide production as well as H_2_O_2_ accumulation is reduced in *rbohd* knockout mutants. In addition, these plants have been shown to be more sensitive to anoxia compared to the wildtype [143,144,145]. Interestingly, RBOHD has been shown to interact with the hypoxia-responsive universal stress protein 1 (HRU1), indicating that HRU1 might function as a hub connecting low O_2_ sensing and ROS signaling [146]. Another important fact of ROS signaling during O_2_ deprivation is that ROS are able to activate the Ca^2+^ channels located in the plasma membrane, slow down anion channels, and inhibit K^+^ influx channels via Ca^2+^, pH as well as nitric oxide (NO) signaling [147,148,149].

A process known as the hemoglobin (Hb)/NO cycle describes an alternative type of respiration via the mtETC under low O_2_ stress [150,151]. Both molecules increase under hypoxic conditions and maintain the ATP gradient by consuming NADH, although the detailed mechanism is unknown so far. However, Hb and NO are involved in the regulation of the energy level in plant cells under low O_2_ conditions. Hence, Hb has been hypothesized to trigger a signaling cascade through ethylene and NO [150]. Increased levels of ethylene as well as NO both promote aerenchyma formation, thereby maintaining the energy and redox status [152]. Furthermore, AOX was shown to prevent NO and superoxide production under normoxia, while superoxide generation was prevented and NO production was increased by AOX under hypoxic conditions [153].

## 4. Hypoxia-Induced Transcriptional Reprograming as a Strategy to Prevent Oxidative Stress

Transcription factors (TFs) play key roles in the cellular adaptation to low O_2_ stress, since many tolerance-mediating genes, including those related to metabolic adjustment, are strongly upregulated upon O_2_ deficiency [154]. The TF families participating in transcriptional reprogramming under hypoxia include group-VII ethylene-response factors (ERF-VIIs) [155,156], no apical meristem (NAM), Arabidopsis transcription activation factor (ATAF), and cup-shaped cotyledon (CUC) (NAC domain proteins) [157,158], WRKY [157,159], HEAT-SHOCK FACTOR (HSF) [160] and MYB [161] factors. ERF-VII factors are currently of the utmost importance and the subject of extensive research. 

### 4.1. Hypoxia-Related Transcription Factors Initiate Gene Expression in Response to Low O_2_ Conditions

**Figure 2:**
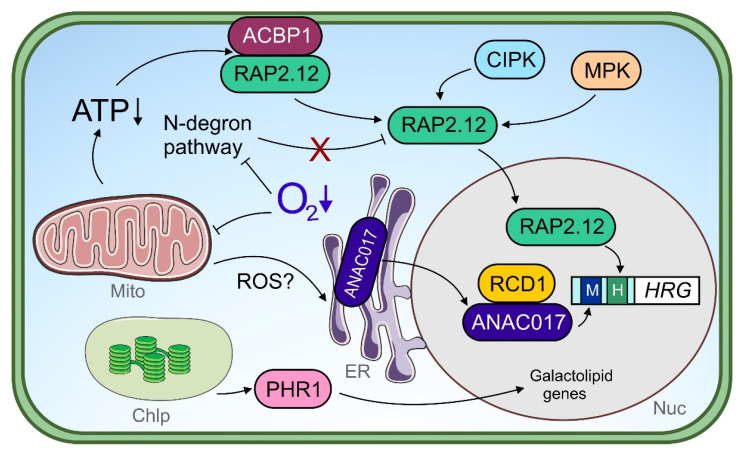
Transcriptional regulators under hypoxia. Under O_2_ limitation, the mitochondrial function is impaired leading to lower ATP production. An energy crisis induces dissociation of the ACBP1-RAP2.12 complex at the plasma membrane. Released RAP2.12 translocates to the nucleus and free RAP2.12 protein is stabilized upon a dysfunctional N-degron pathway, which uses O_2_ as a co-substrate. The post-translational modification of RAP2.12 by mitogen-activated protein kinases (MPKs) and calcineurin-b-like interacting kinases (CIPKs) may regulate transcription factor (TF) function. In the nucleus, RAP2.12 activates hypoxia-responsive genes (HRG) and binds to the hypoxia-responsive promoter element (HRPE, shown as “H”) within target promoters. Endoplasmic reticulum-(ER-)localized ANAC017 is mobilized upon a mitochondrial dysfunction signal under hypoxia, potentially by reactive oxygen species (ROS). Nuclear ANAC017 is regulated in its function via physical interaction with radical-induced cell death 1 (RCD1) and binds to target gene promoters harboring the mitochondrial dysfunction motif (MDM, shown as “M”). Chloroplast signals under hypoxia are suggested to be transmitted to the nucleus by phosphate starvation response 1 (PHR1) and lead to the activation of galactolipid genes. Chlp, chloroplast; ER, endoplasmic reticulum; Mito, mitochondrion; Nuc, nucleus.

ERF-VII family members are found to be conserved within the plant kingdom, and share sequential/structural similarities and a common function under low O_2_ stress in rice [29], barley [162], wheat [163], maize [164], Arabidopsis [155,156] and other species. The conserved N-termini of ERF-VII factors possess a pentultimate cysteine (Cys) residue, rendering some of these TFs substrates of the N-degron pathway. This multi-step enzymatic pathway O_2_-dependently oxidizes the Cys residue and, following other modification steps, such as arginylation and ubiquitination, supplies the protein for proteasomal degradation [156,165,166]. Hence, under aerobic conditions, the ERF-VII protein is degraded constantly, while under hypoxic conditions, where Cys oxidation is hampered, protein stabilization is achieved. This protein degradation mechanism enables the integration of the internal cellular O_2_ concentration into the ERF-VII signaling pathways. However, one has to distinguish between (i) high metabolic activity resulting in low O_2_ levels within plant cells or tissues and (ii) environmental hypoxia, e.g., due to flooding stress, leading to an actual energy crisis for which metabolic adjustment is required. For discrimination, other intracellular signals next to O_2_ availability have to be integrated in order to provoke a cellular reaction tailored to the current (stress) situation [167,168]. Indeed, many cytosolic parameters, such as ATP level and Ca^2+^ level, pH and redox status, change rapidly during hypoxia and have the capacity to participate in signaling processes [116]. In addition, the ethylene formed under hypoxia promotes ERF-VII protein stabilization [169]. The drop in ATP levels under low O_2_ induces the release of plasma membrane-stored RAP2.12, an Arabidopsis ERF-VII factor, and its accumulation in the nucleus (Figure 2). Here, the energy deficiency signal is transmitted via the fatty acid precursor C18:1-CoA, which binds as a specific ligand to the ACBP:RAP2.12 complex at the plasma membrane, resulting in TF dissociation (Figure 2). Thereby, the integration of the actual O_2_ concentration and the energy status within the ERF-VII signaling pathway is achieved [170]. 

Strikingly, various studies in Arabidopsis have demonstrated that ERF-VII factor stabilization and translocation alone are insufficient to activate downstream hypoxia-responsive genes fully [169,170]. Instead, ERF-VII factors are presumably regulated in their activity under hypoxia by additional mechanisms that exceed our current state of knowledge. As one post-translational modification strategy, the phosphorylation of ERF-VII proteins in a non-hypoxic context has been reported, namely, the phosphorylation of RAP2.2 by CIPK11 and CIPK14 and RAP2.3 by MPK3 and MPK6 in Arabidopsis (Figure 2) [171,172]. Remarkably, the MPK-assisted phosphorylation of RAP2.3 at the serine residue at position 151 (Ser-151) results in enhanced TF activity. The ERF-VII factor SUB1A in rice represents another potential target of MPK-dependent modification, as its flooding tolerance-conferring *SUB1A1* allele produces a protein harboring a Ser-186 in a variable region C-terminal to the ERF domain, which constitutes a putative MPK phosphorylation site. Interestingly, this site is not present in the *SUB1A2*-specific TF protein correlating with only moderate flooding tolerance [27]. Moreover, phosphorylation of the *SUB1A1*- but not *SUB1A2*-derived protein by MPK3 was demonstrated [173]. Since CIPK11 and CIPK14 belong to Ca^2+^-regulated kinases, typically activated by intracellular Ca^2+^ influx [174], and MPK3 and MPK6, at least in Arabidopsis, are redox-sensitive proteins directly activated by H_2_O_2_ and induced under O_2_ deficiency upon a mitochondrial ROS signal (Figure 2) [104,175], the action of these different kinase families on ERF-VII factors may represent entry points for multiple Ca^2+^ and ROS signals into the ERF-VII transduction pathway under low O_2_ stress. In turn, ERF-VII factors themselves partially act on kinase gene expression under low O_2_ stress, as shown with RAP2.12 inducing *CIPK25* in Arabidopsis and the *SUB1A1* allele-specific protein inducing *MPK3* expression in rice [173,176].

ERF-VII factors interact with other ERF-VII factors, but also other TF families, to orchestrate hypoxia-responsive gene expression. For instance, SUB1A from rice is capable of inducing two downstream *ERF-VII* genes, *ERF66* and *ERF67,* through direct DNA interaction, thereby forming a transcriptional triad [177]. While SUB1A itself is not a target of the N-degron pathway [165], the protein stability of ERF66 and ERF67 is O_2_-dependently regulated. Thereby, both downstream ERF-VII factors strengthen the transcriptional response to submergence orchestrated by SUB1A [177]. 

LOB domain containing protein 41 (LBD41) is a transcriptional repressor in Arabidopsis [178], whose transcript level is dramatically induced by hypoxia [154]. As *LBD41* shows upregulation in N-degron pathway-deficient mutants under aerobic conditions [165,179], its expression level under hypoxia is clearly ERF-VII-dependent. Furthermore, RAP2.2 and RAP2.12 activate the *LBD1* promoter via the hypoxia-responsive promoter element (HRPE), since a lack of the *cis*-element prevents ERF-VII-mediated *LBD41* responses to hypoxia [180].

A more recent study in Arabidopsis unveiled that WRKY33 and WRKY12 act upstream of ERF-VII-dependent gene expression under hypoxia [159]. Both WRKY factors synergistically activate *RAP2.2* expression under hypoxia by binding directly to the W-box within its promoter, and thereby contribute to submergence tolerance. Interestingly, while three other Arabidopsis *ERF-VII* genes (*RAP2.12*, *HRE1* and *HRE2*) contain such a W-box in their gene-regulatory regions as well, only *RAP2.2* induction by dark submergence appears to be WRKY33- and WRKY12-dependent. *RAP2.2* overexpression in turn could complement the submergence-sensitive phenotypes of *wrky33* or *wrky12* knock-out mutants. That the functions of WRKY and ERF-VII factors under hypoxia are intertwined at multiple levels is, in addition, reflected by the finding that RAP2.2 activates the *WRKY33* promoter directly via an ERF-VII-specific HRPE copy, while RAP2.12 does so in an indirect manner. Thereby, both ERF-VII factors establish a negative feedback loop [159].

RAP2.12 activity is fine-tuned by repressing TF HRA1, whose transcript level is induced upon hypoxia by RAP2.12; thereby, a negative feedback loop is established [181,182]. HRA1 itself is capable of directly binding to hypoxia-responsive target promoters of RAP2.12, including its own endogenous promoter. The authors propose that the physical interaction of HRA1 with target DNA may dampen RAP2.12 activity on precisely that [181]. The action of HRA1 is, interestingly, RAP2.12-specific, and does not cover other ERF-VII factors. It remains open through which molecular mechanism HRA1 is repressing RAP2.12 function and, in case other repressors exist, whether their expression levels depend on ERF-VII factors as well.

### 4.2. Mitochondrial Retrograde Signals Induce Nuclear Gene Expression in Response to Hypoxia

The mitochondrion, as the primary organelle being affected in its function by O_2_ limitation, is capable of inducing nuclear gene expression through retrograde signaling [157,158]. Many hypoxia-induced genes respond to pharmacologically induced mitochondrial dysfunction, such as antimycin-A treatment, including the Arabidopsis *ERF-VII* gene *HRE2* [138], which transcriptionally links mitochondrial retrograde signaling with ERF-VII transduction pathways. Endoplasmic reticulum (ER)-located NAC transcription factors, such as ANAC017 and ANAC013 in particular, facilitate mitochondrial retrograde signals in order to induce nuclear gene expression [183,184]. Under submergence, ANAC017 improves tolerance by assisting in retrograde signaling-coordinated chloroplast function [157]. Notably, in *anac017* knock-out mutants, photosynthesis rate and chlorophyll content were lower and ROS production higher than in the wildtype under submergence followed by reaeration. ANAC017 likely conducts transcriptional reprogramming under low O_2_ stress by inducing other TF genes, including *WRKY40* and *WRKY45* [157]. While the role of both WRKYs in signaling in general and retrograde signaling in particular is largely unexplored under hypoxia, *WRKY40* or *WRKY45* overexpression improved, and their knockout lowered submergence tolerance in Arabidopsis, respectively [157]. Another recent study reports the regulation of mitochondrion-related, ROS-induced genes by ANAC017 under submergence (Figure 2) [158]. Here, the action of ANAC017 is, interestingly, more apparent at younger than at later plant developmental stages, and positively correlates with a higher submergence tolerance in the juvenile phase.

ER-bound NAC factors are presumably cleaved at their transmembrane domain by intramembrane proteases, and the protease activity might be regulated in a mitochondrion-dependent manner [183]. Under non-stress conditions, the ectopic expression of a truncated ANAC017 lacking its transmembrane domain, i.e., the TF is soluble and accumulates in the nucleus even in the absence of a mitochondrial stress signal, leads to elevated transcript levels of multiple anaerobic genes, including *HRE2*, *PDC1* and *PGB1* [185]. This suggests not only an involvement of ANAC017 in hypoxia-responsive gene expression in general, but also underlines the importance of a tightly controlled release of the TF from the ER under stress. The cleavage of ANAC017 under submergence has indeed been observed, but after a 24 h treatment [158], suggesting a role of ANAC017 in rather slow transcriptional responses. Moreover, as antimycin-A treatment promoted ANAC017 cleavage [158] (mitochondrial), ROS signals represent promising candidates for low O_2_ stress-induced ANAC017 release from the ER. Exciting questions still to be answered in terms of mitochondrial retrograde signaling under low O_2_ stress are: (i) which mitochondrial protein(s) initiate(s) retrograde signaling, (ii) which signal(s) mediate(s) the mitochondrion-to-ER transfer, (iii) how is the release of ANAC017 from the ER mechanistically initiated and, finally, what is the role of the ER itself in mitochondrial retrograde signaling? 

ANAC017 is linked to the cellular ROS system by activating ROS-related genes, even under submergence [158], and interacts with ROS-associated proteins [186]. One of these interactors is radical-induced cell death 1 (RCD1), an (ADP-ribosyl) transferase containing the typical poly (ADP-ribose) polymerase (PARP) domain, whose enzymatic function is inactive [186,187]. RCD1 interacts with a variety of TFs [187]; however, it is not understood at all through which molecular mechanism RCD1 interferes with TF function. Remarkably, RCD1 contains seven Cys residues within its interdomain linkers, rendering the protein redox-sensitive. Upon oxidative stress, the oxidation of RCD1 results in its protein decay [186]. It may be speculated that, via the RCD1-ANAC107 module, redox signals generated under low O_2_ stress are integrated into retrograde signaling and affect downstream gene expression (Figure 2). In addition, TFs of the HSF family potentially contribute to redox-dependent hypoxia signaling. HSFA1a, HSFA1b and HSFA2 are well-known positive regulators of Arabidopsis’ low O_2_ stress tolerance [160], of which HSFA1a is activated upon thiol oxidation [188]. Still, redox-dependent signaling under hypoxia is an emerging field and awaits further research effort (reviewed in [189]).

While mitochondrial retrograde signaling is becoming more and more the subject of interest under low O_2_ stress, retrograde signaling events derived from the chloroplast are much less explored. Notably, ANAC017 and its repressor RCD1 together establish a communication link between the chloroplast and the mitochondrion (Figure 2) [186,190], which strengthens the already suggested role of ANAC017 in chloroplast function coordination [157]. The loss of *RCD1* increases the expression of mitochondrial dysfunction stimulon (MDS) genes typically regulated by ANAC017 [186,191]. It would be worthwhile to test whether the ANAC017-RCD1 module also participates in plastid-to-mitochondrion communication pathways during low O_2_ stress. Under hypoxia, the *rcd1* mutant shows an impressive tolerance towards methyl viologen (MV), which leads to ROS formation in the chloroplast in the light [190]. This enhanced phenotype is interestingly not the consequence of altered plastidial ROS scavenging abilities, but can be traced back to a disrupted electron transfer from photosystem I (PSI) to MV found in the wildtype. Likewise, a correlation between improved MV tolerance under aerobic conditions and enhanced MDS expression is observed for *ANAC017* overexpression plants [191]. It is worth mentioning in the context of illuminated hypoxia-specific chloroplast signaling the MYB factor phosphate starvation 1 (PHR1), which mediates both transcriptional responses to O_2_ deficiency and phosphate starvation, and is potentially post-translationally activated by a retrograde signal upon lowered photosynthesis (Figure 2). Interestingly, PHR1, which mainly regulates galactolipid-related genes (present in chloroplast membranes) but not anaerobic core genes, acts completely independently of ERF-VII factors or the N-degron pathway under hypoxia [161]. 

### 4.3. Gene Regulatory Mechanisms and Chromatin Modifications during O_2_ Deprivation

The upstream regulation of ERF-VII factors and other TFs under low O_2_ stress is the subject of extensive ongoing research, but less light is shed on the downstream mechanisms of hypoxia gene induction. The process of transcription initiation typically involves recruitment of the general transcriptional machinery, i.e., general TFs and RNA polymerase II (RNAPII) [192] via multiple co-factors. Remarkably, ANAC017 has been linked mechanistically to regulator of alternative oxidase 1 (RAO1), also known as cyclin-dependent kinase e1 (CDKE1), which is a kinase unit of the Mediator complex [193]. The Mediator complex is conserved among eukaryotes and functions as a bridge between (stress-related) TFs and the general transcriptional machinery of the cell [192]. The flooding-sensitive phenotype of the *rao1* knock-out mutant suggests a regulatory role of the Mediator complex under low O_2_ stress [157]. Moreover, as CDKE1 interacts with sucrose non-fermenting related kinase 1 (SnRK1/KIN10), it integrates information on the cellular energy status into mitochondrial retrograde signaling, at least under non-stress conditions [193]. Whether the employment of the Mediator complex in gene regulation under low O_2_ stress covers only mitochondrial retrograde signaling-related TFs such as ANAC017, or is extended to other important TF families, has yet to be explored. This assumption is, however, likely, since RAP2.2 as an ERF-VII representative interacts with the Mediator subunit MED25 in a non-hypoxia context [194]. 

Interestingly, a proportion of anaerobic core genes in Arabidopsis, including *LBD41*, *PGB1* and *PCO2*, rapidly respond to hypoxia within minutes of exposure. Thereby, genes respond earlier than ERF-VII factors appear in the nucleus (after 3 hours of hypoxia) [195]. While these findings are solely based on a single ERF-VII factor—RAP2.12—and need further exploration, the possibility exists that those rapidly responding transcripts, at least in the first three hours of stress, are not induced by ERF-VII factors, but by early-acting, so far unidentified TFs. 

Hypoxia not only stimulates the transcription and translation of anaerobic core genes, but also translationally blocks unrelated transcripts that are either subjected to degradation or kept in stress granules for further storage [196]. Preferentially non-uracil-rich mRNA is bound by oligouridylate binding protein 1 (UBP1) under hypoxia, and its aggregation results in granule formation. By the discrimination between hypoxia-related and -unrelated transcripts, an energy-saving adaptation strategy is followed under O_2_ constraints, and *UBP1* knockout seedlings are consistently impaired in low O_2_ tolerance. Notably, while transient hypoxia promotes (UBP1-dependent) stress granule formation, re-oxygenation is accompanied by a concomitant decay of UBP1-stress granules [196]. The production but also autophagy of stress granules specific to hypoxia is furthermore regulated in a Ca^2+^-dependent manner, and involves the Ca^2+^ sensor CALMODULIN-LIKE 38 (CML38) [197]. CML38 was found to co-localize with the mRNP stress granule marker RNA Binding Protein 47 (RBP47), and furthermore interacts with proteins associated with messenger RNA ribonucleoprotein (mRNP) complexes. The stress granule disorganization in *cml38* mutants points to the multiple functions of CML38 in granule formation, maintenance and degradation under long-term hypoxia, and, in addition, indicates that CML38 promotes increased autophagy during re-oxygenation [198]. 

The accessibility of regulatory target DNA to stress-related TFs, as well as those acting under hypoxia, is mandatory in order to induce gene expression upon stress stimulus. Chromatin modifications and DNA methylation are important aspects of gene regulation under stress. Under submergence, the occurrence of repressing histone modifications, and thus a lower chromatin accessibility, in ANAC017-regulated promoters correlates with decreased submergence tolerance in older plant stages [158], indicating that epigenetic effects may represent an important aspect of low O_2_ adaptation in plants. Indeed, transcription-activating histone 3 (H3) Lysine 4 trimethylation (H3K4me3) has been reported under submergence for the two hypoxia-responsive promoters *PDC* and *ADH* in rice [199]. A recent study in Arabidopsis on chromatin modification patterns under hypoxia revealed that the gene-regulatory regions of strongly hypoxia-responding genes are exempted from the transcription-repressing Histone 2A variant H2A.Z and, in addition, show Histone 3 modifications, such as H3 Lysine 9 acetylation (H3K9ac), but also H3K4me3, both promoting transcription [200]. Interestingly, the removal of H2A.Z is partially ERF-VII-dependently regulated, as multiple ERF-VII factors physically interact with BRAHMA, an H2A.Z-related chromatin remodeling ATPase, and thereby oppose its function [201,202]. 

To date, chromatin-modifying components largely await identification under hypoxia. In this context, it may be helpful to consider potential similarities between hypoxia and other stresses. For instance, temperature stress, similar to hypoxia, promotes the reduced association of the repressing histone variant H2A.Z prior to the transcriptional activation of heat-responsive genes [203]. The O_2_-sensing mechanism provided by the N-degron pathway directly accesses epigenetic gene repression by regulating the stability of polycomb repressor complex 2 (PRC2) subunit vernalisation 2 (VRN2) [204]. VRN2 protein abundance accumulates under both hypoxia and cold stress, and, likewise, anaerobic core genes show overlapping transcriptional responses to both stresses, hinting at similar epigenetic regulatory mechanisms under low O_2_ and low temperatures. VRN2 may have an exciting role in stress memory under hypoxia, as it does under cold stress, where a continuously stabilized VRN2 protein keeps the PRC2 complex active under extended cold periods and induces flowering when warmer temperatures arise [204]. It would be highly interesting to explore in detail the phenomenon of stress memory under O_2_ constraints, and to investigate to what extent VRN2, but also other epigenetic regulators, participate in it.

## 5. Post-Translational Modifications Enable a Rapid and Fine-Tuned Response to Hypoxia

Under hypoxic conditions, the COX pathway is inhibited, while glycolysis, fermentation and the GABA shunt are stimulated (Figure 1B). This leads to the accumulation of various metabolic intermediates such as succinate, citrate and malate [205,206]. On the transcriptional level, citrate has been shown to induce Arabidopsis *AOX1A* expression [206]. In addition, several studies have shown that the AOX amount can change in response to hypoxia, anoxia, or re-oxygenation after low O_2_ treatment [207,208,209,210,211,212]. Jayawardhane et al. [153] also demonstrated that AOX was particularly important in preventing nitro-oxidative stress during re-oxygenation.

While the post-translational regulation of AOX activity relies on redox mechanisms and the formation of a thiohemiacetal at CysI with 2-oxo acids in most plant species, in some instances, AOX isoforms can be stimulated by succinate, possibly through the formation of an ester bond [213,214,215,216,217,218,219]. Succinate activation of AOX isoforms relies on the presence of a Ser residue at the position of CysI, and leads to insensitivity to 2-oxo acid activation. However, *Oryza sativa* (rice), *Nelumbo nucifera* (lotus), *Zea mays* (maize), *Solanum lycopersicum* (tomato) and *Chlamydomonas reinhardtii* naturally possess AOX isoforms containing a Ser residue at the position of CysI (for alignment see [218]). The succinate-dependent post-translational activation of AOX isoforms possessing the Ser residue at the position of CysI may allow for an increased capacity of the AOX pathway, and may enable plants to survive during hypoxic conditions. This mechanism might even play an important role in adaptation to wet or flooded habitats, as well as recovery following reaeration. Furthermore, the tight cuticle of tomato fruits and maize kernels leads to hypoxic metabolism in these organs, additionally pinpointing the hypothesized role of succinate activation of AOX isoforms under hypoxic conditions. In addition to the potential role of succinate in AOX activation, the succinylation of TCA cycle and glycolytic enzymes has been suggested as an important regulatory mechanism to control metabolic processes under low O_2_ conditions [138,220]. Therefore, succinate accumulation might play a key role under limited O_2_ and post-hypoxic conditions to guarantee plant survival. Similar to other post-translational modifications, such as arginylation and ubiquitination (ERF-VII factors), phosphorylation and protein acetylation critically rely on substrates that can be derived from mitochondrial energy metabolism and are therefore involved in the regulation of plant metabolism under limiting O_2_ conditions.

There is a big gap in our knowledge about post-translational modifications in other cell compartments that might contribute to the plant stress response to O_2_ deprivation. However, using a redox sensor such as the redox-sensitive green fluorescent protein 2 (roGFP2), Wagner et al. [116] demonstrated that low O_2_ stress leads to a strong transition from reducing to oxidizing conditions in the cytosol. There is increasing evidence demonstrating that cytosolic enzymes of the central metabolism, such as glyceraldehyde 3-phosphate dehydrogenase, MDH, enolase, LDH and isocitrate dehydrogenase, are able to change their properties in the oxidized and post-translationally modified form, translocate to other subcellular compartments and take over new tasks (for review see [221]). These so-called moonlighting functions directly link energy metabolism to adaptive stress responses that are required for the maintenance of redox homeostasis and rapid responses that might also be of high importance during low O_2_ conditions and re-oxygenation. However, this hypothesis needs experimental proof and should be assessed in the future.

## 6. Re-Oxygenation—A Challenge of Plant Survival Post-Hypoxic Conditions

**Figure 3:**
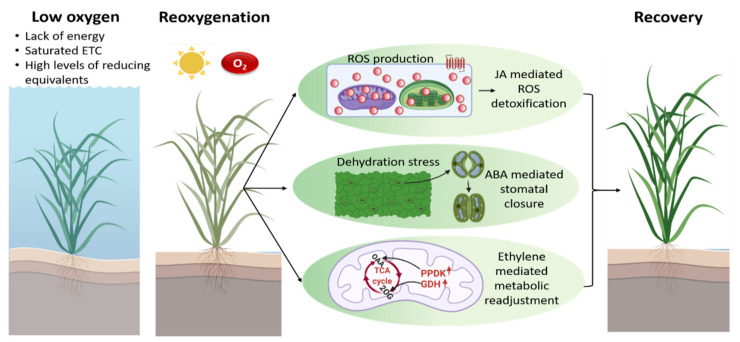
Under water, plants experience a shortage in O_2_ that ultimately affects the mtETC. Conditions such as a lack of energy, saturated mtETC and high levels of reducing equivalent prevail during hypoxia. A shift to normal environmental conditions with intense light and higher O_2_ after flood water retreats results in excess ROS generation in chloroplasts and mitochondria, respectively. On top of that, plants also encounter dehydration stress and an energy crisis immediately post-submergence. The re-oxygenation response is mediated via JA, ABA and ethylene signaling to counteract excess ROS generation, to overcome dehydration stress and to generate energy through metabolic readjustment. 2-OG, 2-oxoglutarate; GDH, glutamate dehydrogenase; OAA, oxaloacetate; PPDK, pyruvate phosphate dikinase. The figure was created using BioRender.com, accessed on 10 January 2022.

Since flooding is a biphasic stress, with an initial reduction of molecular O_2_ during submergence followed by re-oxygenation post-flooding, re-exposure of plants to normoxic conditions imposes more challenges on the plant, which may be as severe as or even more severe than the O_2_ deficiency itself [42]. During submergence, plant tissues adapt to low O_2_ and low light in turbid water, while re-exposure to atmospheric O_2_ after the low O_2_ phase results in post-anoxic injuries, specifically due to the excess generation of ROS [157,222,223]. Prolonged hypoxia or anoxia during submergence leads to a saturated mtETC, low adenylate energy charge, and high levels of reducing equivalents (Figure 3), which can have a direct effect on intracellular ROS production once O_2_ becomes available during re-oxygenation [224]. Re-oxygenation restores aerobic metabolism in plant cells to meet the high energy demand. However, the accelerated activity of mitochondrial respiration additionally stimulates the generation of ROS [225,226,227,228].

## 7. Plants Have to Cope with Several Injuries Occurring Post-Hypoxic Conditions

### 7.1. Oxidative Stress Is Increased during Reaeration

Post-hypoxic injuries are mainly associated with the excess generation of ROS leading to the oxidation of lipid membranes, proteins, nucleic acids, and carbohydrates [40,229]. Besides peroxisomes, chloroplasts and mitochondria are the primary sites of ROS production in photosynthetically active tissues, predominantly due to their high metabolic activities and increased rate of electron transfer. The PSI and PSII reaction centers are major sites of ROS production in the chloroplast [230,231], while complex I and III of the mtETC are major sites for mitochondrial superoxide (O_2_^−^) production. Roughly 2–5% of the total O_2_ consumed by mitochondria is converted to O_2_^−^ [232]. In Arabidopsis, reaeration induces *RBOHD* transcripts, which results in the generation of O_2_^−^ in the apoplastic space [223]. In the wetland species *Alternanthera philoxeroides*, increased abundance of ROS-generating acetaldehyde was observed after desubmergence [233]. In addition, increased generation of ROS radicals was observed in Arabidopsis leaves within 3 h of exposure to post-hypoxic conditions [223]. The roots of lupine (*Lupinus luteus*) exposed to a hypoxia–re-oxygenation regime caused an increase in the level of free radicals and H_2_O_2_, which confirms an increased ROS production during re-oxygenation. Re-oxygenation also triggered a higher activity of superoxide dismutase (SOD) and catalase (CAT) in roots of lupine [227]. Furthermore, increased malondialdehyde (MDA) content as a consequence of ROS-mediated lipid peroxidation has been observed post-hypoxic conditions in Arabidopsis [223,234], rice seedlings [41,235], and soybean [236]. Excess accumulation of ROS during reaeration is a combinatorial effect of increased ROS generation due to an increased electron pressure in the chloroplast and mitochondrial electron transport chains, as well as a result of reduced scavenging capacity [237,238].

### 7.2. Photoinhibition Results from Increased Light Intensities Experienced Post-Submergence

Submergence results in the severe loss of carbon reserves required to meet the physiological energy demand and limited light conditions. Furthermore, limited CO_2_ diffusion reduces photosynthesis and the restoration of carbon reserves. However, reaeration leads to a sudden increase in O_2_ concentrations and a simultaneous increase in light intensity due to the outflow of water. In particular, the shift from low to high light conditions experienced by plants during re-oxygenation can lead to phototoxic damage at PSII, resulting in the inactivation of the reaction centers [239]. In *Hemarthria altissima, A. philoxeroides* and rice, a considerable decline in the maximum quantum yield of PSII (F_v_/F_m_) and nonphotochemical energy quenching (NPQ) could be observed during desubmergence [235,240,241]. Furthermore, a significant reduction in F_v_/F_m_ was observed after 5 h post-hypoxia in Arabidopsis accessions [223]. In the grass species *Agrostis stolonifera*, *Cynodon dactylon*, and *Zoysia japonica*, reduced F_v_/F_m_ values could be observed after 1 day of re-oxygenation [242]. Therefore, recovery from PSII photodamage is essential to restore submergence-depleted energy reserves, which in turn prevent leaf senescence and cell death [243].

### 7.3. Fine-Tuning of Stomatal Conductance Is Essential during Re-Oxygenation

A decrease in water conductivity and desiccation stress on desubmergence was observed despite the soil being soaked with water [41]. Submergence and reaeration affect the optimal function of roots due to decreased root hydraulic conductance, which reflects the ability of roots to absorb water [244,245]. Dehydration leads to a decreased relative water content (RWC), loss of leaf turgidity, leaf rolling, and wilting [41,223,235,246]. Therefore, ABA-mediated stomatal closure plays an important role to prevent transpirational water loss in leaves during recovery (Figure 3) [223,235]. In the intolerant Arabidopsis accession Bay-0 and rice cultivar M202, severe dehydration symptoms have been observed due to decreased stomatal conductance and the inability to close the stomata [41,223,234]. However, plants must keep a fine balance between the gradual opening and closing of the stomata, since closed stomata also result in limited CO_2_ uptake, which in turn affects photosynthesis and recovery.

### 7.4. Plants Accumulate Various Toxic Metabolites during Reaeration

In addition to factors such as excess ROS generation caused by re-oxygenation, re-illumination, and water loss, plants also accumulate toxic compounds and show lipid peroxidation during submergence and desubmergence [225]. The roots of wheat and rice seedlings showed increased lipid peroxidation (LPO) and accumulation of end-LPO products during re-oxygenation after prolonged anaerobiosis [225,247]. Moreover, the accumulation of diene and triene conjugates of fatty acids (primary intermediates of LPO) and thiobarbituric acid (TBA)-reactive end products of LPO was observed in roots of sensitive *Iris germanica*, *Triticum aestivum* and *Avena sativa* [248]. Similarly, intensive lipid hydrolysis was observed, along with the accumulation of LPO products, during re-oxygenation in potato cells and potato plants [226,249]. The tubers of sensitive potato plants accumulated 5–7-fold increased acetaldehyde during re-oxygenation [46].

### 7.5. Senescence Is Induced under Post-Hypoxic Conditions

Energy reduction, cytoplasmic acidosis, electron leakage and ROS production, moisture loss, and the accumulation of toxic products of anaerobic metabolism together significantly impair plant survival, contributing to post-anoxic damage and leading to programmed cell death or necrosis [236,250]. Senescence usually happens to be the last phase of leaf development and is typically characterized by leaf yellowing through chlorophyll degradation [251,252,253]. In the intolerant Arabidopsis accession Bay-0, desubmergence induced the expression of NAC domain-containing protein6/Oresara1 (ORE1) [223], a positive regulator of leaf senescence [254,255,256]. In rice, the presence of the *SUB1A* loci significantly reduces chlorophyll breakdown during submergence [26]. The retention of chlorophyll was observed in the forage grass *Melilotus siculus* simultaneously with senescence avoidance in submergence recovery [257].

## 8. Re-Adjustment of Plant Metabolism Is Initiated during Re-Oxygenation

A significant decrease in ATP production via mtETC [138,258] is probably one of the most influential metabolic processes for successive re-oxygenation survival. Decreased ATP is linked to increased cytoplasmic acidity, potentially hindering recovery upon re-oxygenation [53]. Various metabolic processes such as glycolysis, fermentation and TCA cycle replenishment were fine-tuned during re-oxygenation. While hypoxia results in the accumulation of alanine, re-oxygenation leads to the production of pyruvate and NADH that can be directed to the TCA cycle. During re-oxygenation, ethylene signaling mediated by ethylene-insensitive 3 (EIN3) enhances the expression of glutamate dehydrogenase (GDH) that catalyzes the interconversion of glutamate and 2-OG (Figure 3) [258]. Via the transaminase reaction, 2-OG reacts with alanine to allow the refueling of pyruvate in the TCA cycle. Interestingly, it has been reported that the absence of GDH results in the disturbance of carbohydrate metabolism, phytosterol biosynthesis, and energy regeneration. α-Tocopherol and phytosterols are vital components that are needed for the stability of cell membranes [259,260,261]. Phytosterols are known to maintain membrane integrity and fluidity in response to large temperature variations [262]. Hence, it has been suggested that re-oxygenation results in the activation of a membrane repair system that incorporates newly synthesized α-tocopherol and phytosterols to strengthen damaged membranes [258,263]. In addition, the enzyme pyruvate phosphate dikinase (PPDK) is regulated by ethylene during re-oxygenation (Figure 3). The PPDK enzyme catalyzes the conversion of phosphoenolpyruvate to pyruvate, which is further converted to OAA. Therefore, ethylene overall supports TCA cycle replenishment and carbohydrate metabolism during re-oxygenation [258,263]. 

Ethanol that has been produced during low O_2_ conditions is oxidized to acetaldehyde during re-oxygenation. Acetaldehyde is then further metabolized by aldehyde dehydrogenase to aid plant recovery [264]. The accumulation of metabolites such as arabinose and trehalose during re-oxygenation has also been reported [55,265]. Furthermore, plant recovery after hypoxia or anoxia also involves several other mitochondrial metabolic processes, such as polyamine production based on basic amino acid metabolism, respiratory chain function, and alternative respiration. In addition, changes in the morphology of plant mitochondria under hypoxia or anoxia and re-oxygenation are well documented by Shingaki-Wells et al. [250]. There is also evidence of mitochondria disintegration in the absence of O_2_ in anoxia-intolerant wheat [85]. However, anoxia-tolerant rice and *Echinochloa phyllopogon* were able to maintain the ultrastructure and shape of their mitochondria [266]. Besides changes in ultrastructure, mitochondrial ROS/RNS generation is a key element in anoxia and re-oxygenation stress signaling. The involvement of respiratory chain components in NO and ROS signaling under low O_2_ has also been reported [225,267]. Thus, apart from playing a major role in energy production, the mtETC could play a signaling role in anoxic and post-anoxic survival. Mitochondria—and especially its alternative pathways—have been shown to be involved in NO homeostasis during hypoxia [268]. As part of the alternative electron transport pathway, AOX aids nitrite-dependent NO production in mitochondria during hypoxia. However, under normoxia, AOX minimizes NO synthesis, ROS generation and peroxynitrite formation [269]. Therefore, AOX might function as a crucial switch in the hypoxia–re-oxygenation transition by regulating ROS/RNS-dependent protein modifications during re-oxygenation. Recently, the role of AOX in preventing nitro-oxidative stress during the re-oxygenation period has been reported, which allows for the recovery of the energy status following hypoxia [153]. 

The alternative electron transport pathway components, AOX and NDs, provide high flexibility to plant mitochondria [270]. Alternative mtETC components enhance the electron flow from reducing equivalents generated via the TCA cycle and photosynthesis to O_2_, specifically when the COX pathway is inhibited by abiotic or biotic stresses. Thus, the alternative mtETC components can counteract excess ROS production during re-oxygenation by the fast removal of excess reductive power build-up during hypoxia. At the same time, alternative mtETC components enable the avoidance of oxidative stress, since these components function as a safety valve preventing the excess generation of ROS [271,272]. It has been reported that AOX and NDs are upregulated at the transcriptional, protein, and activity levels when the basal mtETC is compromised [273,274,275]. Hence, alternative mtETC components are likely to play a vital role during re-oxygenation and adaptation. However, further research is needed to prove their function during hypoxic stress and re-oxygenation.

## 9. Survival Strategies of Plants during Re-Oxygenation

One of the primary survival strategies of plants during recovery from anoxia or hypoxia is the upregulation of antioxidative defense mechanisms to minimize the occurrence of oxidative stress. There have been several reports of different plant species upregulating their antioxidative defense system during re-oxygenation [234,236,263]. In Arabidopsis, a high level of ascorbate and glutathione levels was observed in genotypes exhibiting better recovery during re-oxygenation [223,234]. In soybean roots and the hypocotyl, the enzymatic activities of peroxidases were suppressed during flooding, but significantly increased during recovery [276,277]. *Alternanthera philoxeroides* is a submergence-resistant wetland species that uses the escape strategy during submergence via rapid stem elongation. Upon re-oxygenation, *A. philoxeroides* leaves exhibit increased ascorbic acid, CAT, and SOD activities that are indispensable for ROS scavenging [278]. The M202 (Sub1) rice cultivar containing the *SUB1A* submergence-tolerance gene displayed reduced ROS accumulation and less lipid peroxidation upon re-oxygenation [41]. Chickpea seedlings treated with ascorbic acid showed improved plant survival during anoxia and re-oxygenation [279]. Furthermore, a gradual increase in SOD activity was observed in the rhizomes of *Iris pseudacorus* during re-oxygenation compared to the normoxic control. Moreover, the tolerant *I. pseudacorus* showed a higher activity of SOD in comparison to sensitive *I. germanica* [280].

Jasmonic acid (JA) signaling is involved in maintaining the oxidative stress response during re-oxygenation (Figure 3). Re-oxygenation results in the induction of JA biosynthesis genes and the accumulation of JA in Arabidopsis rosettes within a few hours. Mutants deficient in JA signaling or biosynthesis exhibited a weak recovery phenotype. Moreover, the pre-treatment of JA prior to submergence results in better survival after re-oxygenation. The JA-driven re-oxygenation response is under the control of MYC2, a basic helix–loop–helix leucine-zipper TF that promotes the expression of genes involved in ascorbate and glutathione biosynthesis (Figure 3) [234]. Soybean roots treated with JA showed better ROS detoxification and recovery by promoting enzymes involved in nucleotide metabolism [254]. Post-submergence recovery requires a fine-tuned balance between ROS detoxification and alleviation. Since ROS detoxification is important during recovery, complete ROS elimination is harmful for plants, which points towards a signaling role for ROS during reaeration. In this context, it is worth mentioning that the recovery of Arabidopsis plants was hindered when ROS production was significantly restrained upon desubmergence [223]. *SUB1A* transcripts were also substantially induced in response to MV treatment [41]. Hence, the initial ROS burst post-submergence could act as signal for the transition to re-oxygenation and allow plants to trigger adaptive stress responses.

A better antioxidant defense or enhanced ROS scavenging upon re-oxygenation might also be useful for photosynthesis recovery. Efficient ROS scavenging reduces the damage of PS II, which in turn limits photoinhibition and allows for the faster refueling of energy reserves through photosynthetic carbon fixation during recovery. In comparison to the submergence-sensitive Arabidopsis accession Bay-0 and submergence-sensitive M202 in rice, the tolerant accession Lp2-6 and M202 (Sub1) exhibited an optimal F_v_/F_m_ ratio after re-oxygenation [223,235]. Therefore, the replenishment of energy reserves through reactivated photosynthesis during desubmergence is vital for recovery. A correlation is shown between post-submergence carbohydrate status, restoration of photosynthetic ability and flooding tolerance [3,281]. Interestingly, resistant species were able to maintain the functionality of PSII complexes even after long periods of O_2_ deficiency or darkness [26,233,251]. 

Besides ROS detoxification, the dehydration of areal tissues is another key issue in post-hypoxic stress recovery. Plants induce dehydration-responsive genes to maintain osmotic homeostasis and water loss and enhance the survival of shoots. Shoot ABA levels and ABA-and drought-responsive transcripts increased during desubmergence in Arabidopsis and SUB1A rice [41,223,234]. The submergence-sensitive accession Bay-0 in Arabidopsis showed rapid water loss and leaf dehydration even though shoot ABA levels were high. Bay-0 shoots also showed higher ethylene release upon desubmergence, which countered ABA-mediated stomatal closure through the induction of senescence-associated gene 113 (SAG113), resulting in a higher water loss (Figure 3) [223]. Ethylene also enhanced *ORE1* during desubmergence, which directs chlorophyll breakdown. Correspondingly, the knockout mutants *sag113* and *ore1* showed reduced water loss and increased chlorophyll levels, respectively, in comparison to wildtype plants during desubmergence. Similar results were obtained by blocking ethylene perception during recovery [223]. Hence, ethylene induction post-submergence induces senescence and reduces survival in the sensitive Arabidopsis Bay-0 accession [223]. 

There are still several open questions about the role of ethylene during re-oxygenation. Ethylene signaling during re-oxygenation is also required for replenishing TCA cycle intermediates [263]. Interestingly, metabolome studies show that *SUB1A* was induced by ethylene in rice, which alters the TCA cycle flux by stimulating free amino acid synthesis during low O_2_ conditions, which is then quickly overturned on desubmergence [282,283]. It might be possible that the post-submergence response is mediated by hormonal crosstalk between ethylene and JA (Figure 3). *MYC2* overexpressors are known to have enhanced JA signaling and show reduced leaf withering during reaeration, indicating an important role of JA in dehydration regulation. Moreover, DNA-binding activity and downstream ethylene signaling by the TFs EIN3 and EIN3-Like1 (EIL1) are inhibited by MYC2. In *Rumex palustris* and *Rumex maritimus*, JA could alter EIN3 and EIL1 activity to regulate ethylene-mediated re-oxygenation responses [284]. Hence, the re-oxygenation response in Arabidopsis could be maintained by fine-tuning ethylene and JA, and consequently by balancing oxidative stress and energy metabolism [42].

Apart from submergence, recovery post-submergence is also an important aspect of flooding tolerance. Thus, careful investigation of the post-hypoxic recovery phase at the transcriptomic, proteomic and metabolic levels will help us to understand signals and downstream responses to reveal effective recovery strategies. Post-submergence injuries are characterized by excess ROS generation in mitochondria due to the intensive activation of the mtETC in the presence of O_2_ and in chloroplasts due to photoinhibition. Besides this, reduced carbohydrate replenishment, dehydration stress, and senescence also contribute to post-submergence injury. Plants employ strategies such as upregulating ROS-scavengers, antioxidative enzymes and drought-responsive genes to overcome oxidative and dehydration stress. Effective recovery additionally requires an adequate balance in crosstalk between the plant hormones JA and ethylene. However, only a few studies on adaptation and acclimation to the post-hypoxic phase have been made available so far, especially in comparison to studies on the hypoxic or anoxic phase. Although the major signals and factors involved in the regulation of re-oxygenation have been identified, knowledge about the molecular mechanisms directing efficient post-submergence recovery is still very minimal. In order to strengthen our understanding of these processes, along with comparative studies of species with varying degrees of resistance to re-oxygenation damage, the involvement of phytohormones, TFs, and other regulatory responses is necessary.

## Abbreviations

2-OG	2-oxoglutarate
2-OG DH	2-oxoglutarate dehydrogenase
ADH	alcohol dehydrogenase
ALAT	alanine aminotransferase
AOX	alternative oxidase
ATAF	Arabidopsis transcription activation factor
Ca^2+^	calcium
CaM	calmodulin
CAT	catalase
CDKE	cyclin-dependent kinase e
CIPK	calcineurin-b-like interacting kinase
CML	calmodulin-like
CoA	coenzyme A
COX	cytochrome c oxidase
CUC	cup-shaped cotyledon
Cys	cysteine
cyt c	cytochrome c
EIN	ethylene insensitive
ER	endoplasmic reticulum
ERF-VII	group-VII ethylene-response factor
F_v_/F_m_	maximum quantum yield of PSII
GABA	γ-aminobutyric acid
GABA-T	GABA-transaminase
GapC	cytosolic glyceraldehyde-3-phosphate dehydrogenase
GDH	glutamate dehydrogenase
glutamate DC	glutamate decarboxylase
H3	histone 3
H_2_O_2_	hydrogen peroxide
Hb	hemoglobin
HRG	hypoxia-responsive genes
HRPE	hypoxia-responsive promoter element
HRU	hypoxia-responsive universal stress protein
HSF	heat-shock factor
IMM	inner mitochondrial membrane
IMS	intermembrane space
isocitrate DH	isocitrate dehydrogenase
JA	jasmonic acid
LBD	LOB domain containing protein
LDH	lactate dehydrogenase
LPO	lipid peroxidation
MDA	malondialdehyde
MDH	malate dehydrogenase
MDM	mitochondrial dysfunction motif
MDS	mitochondrial dysfunction stimulon
MPK	mitogen-activated protein kinase
mRNP	messenger RNA ribonucleoprotein
mtETC	mitochondrial electron transport chain
MV	methyl viologen
NAM	no apical meristem
NDin/ex	internal/external alternative NAD(P)H dehydrogenase
NO	nitric oxide
NPQ	nonphotochemical energy quenching
O_2_	oxygen
O_2_^−^	superoxide
OAA	oxaloacetate
OMM	outer mitochondrial membrane
ORE	oresara
OXPHOS	oxidative phosphorylation
PARP	poly (ADP-ribose) polymerase
PDC	pyruvate decarboxylase
PGK	phosphoglycerate kinase
PGM	phosphoglycerate mutase
PHR	phosphate starvation response
PPDK	pyruvate phosphate dikinase
PRC	polycomb repressor complex
PSI/PSII	photosystem I/II
pyruvate DH	pyruvate dehydrogenase
RAO	regulator of alternative oxidase
RBOH	respiratory burst oxidase homolog
RBP	RNA binding protein
RCD	radical-induced cell death
RNAP	RNA polymerase
RNS	reactive nitrogen species
roGFP	redox-sensitive green fluorescent protein
ROS	reactive oxygen species
RWC	relative water content
SAG	senescence associated gene
Ser	serine
SnRK	sucrose non-fermenting related kinase
SOD	superoxide dismutase
SSA	succinic semialdehyde
SSADH	succinic semialdehyde dehydrogenase
succinate DH	succinate dehydrogenase
TBA	thiobarbituric acid
TCA	tricarboxylic acid
TF	transcription factor
UBP	oligouridylate binding protein
UQ	ubiquinone
VRN	vernalization
